# Role of N-linked glycosylation in the secretion and enzymatic properties of Rhizopus chinensis lipase expressed in Pichia pastoris

**DOI:** 10.1186/s12934-015-0225-5

**Published:** 2015-03-21

**Authors:** Min Yang, Xiao-Wei Yu, Haiyan Zheng, Chong Sha, Caifeng Zhao, Meiqian Qian, Yan Xu

**Affiliations:** The Key Laboratory of Industrial Biotechnology, Ministry of Education, School of Biotechnology, Jiangnan University, 1800 Lihu Avenue, Wuxi, 214122 Jiangsu China; State Key Laboratory of Food Science and Technology, Jiangnan University, 1800 Lihu Avenue, Wuxi, 214122 Jiangsu China; Biological Mass Spectrometry Facility at Robert wood Johnson medical school and Rutgers, the state university of new jersey, Piscataway, NJ 08854 USA

**Keywords:** N-glycosylation, *Pichia pastoris*, *Rhizopus chinensis* lipase, Enzyme activity, Secretion, Thermostability, LC-MS/MS

## Abstract

**Background:**

The methylotrophic yeast, *Pichia pastoris*, is widely used as a useful experimental tool in protein engineering and production. It is common for proteins expressed in *P. pastoris* to exhibit N-glycosylation. In recent years, glycosylation studies in *P. pastoris* have attracted increasing attention from scholars. *Rhizopus chinensis* lipase (RCL) is one of the most important industrial lipases, and it has four potential N-linked glycosylation sites. The aim of the present study was to determine whether RCL undergoes asparagine-linked (N-linked) glycosylation and to examine the role of this modification in RCL expression and function.

**Results:**

In this study, we demonstrated that RCL expressed in *Pichia pastoris* was N-glycosylated at the sites N-14, N-48 and N-60. The majority of the sites N-14 and N-60 were glycosylated, but the glycosylation degree of the site N-48 was only a very small portion. The glycan on N-60 played a key role in the expression and secretion of RCL. RT-PCR results showed that the mRNA level of *proRCLCN60Q* remained unchanged even though the protein secretion was hampered. Although the N-glycan on N-14 had no effect on the secretion of RCL, this glycan was beneficial for the lipase catalytic activity. On the other hand, the little amount of N-glycan on N-48 had no effect both on the secretion and activity of RCL in *P. pastoris*. Moreover, the thermostability analysis of RCL revealed that the lipase with more N-glycan was more thermostable.

**Conclusions:**

RCL was N-glycosylated when expressed in *P. pastoris*. The N-glycans of RCL on the different sites had different functions for the secretion and enzymatic properties of the lipase. Our report may also provide theoretical support for the improvement of enzyme expression and stability based on the N-linked glycosylation modification to meet the future needs of the biotechnological industry.

## Background

*Pichia pastoris* is a widely used industrial methylotrophic yeast that has been developed as a useful experimental tool in protein engineering and production [1,2]. It is well known that *P. pastoris* can N-glycosylate proteins via mannose oligosaccharide linked to asparagine through two N-acetylglucosamines [3]. Glycosylation is the most common and important form of post-translational modification [4]. The addition of a large glycan attached to the protein backbone can dramatically alter the structure, and consequently the function of the polypeptide architecture [5]. In recent years, glycosylation studies in *P. pastoris* have attracted increasing attention from scholars. The diverse roles of glycans were implicated in the control of the conformational maturation, activity and stability of glycoproteins [6-9].

Since control over the resulting glycan structural and spatial arrangement is believed to be one of the biggest challenges to the study and biomedical employment of glycoproteins [10], a multitude of alternative synthetic methods have been developed in recent years for the production of chemically glycosylated proteins [11,12]. However, most of the scientific insights concerning the effects of glycans on protein biophysics have been derived from the study of the genetic glycosylated proteins [13,14].

Lipases are well known hydrolases capable of hydrolyzing the ester bonds of water-insoluble substrates at the interface between substrate and water, which show remarkable levels of activity and stability in non-aqueous environments, in contrast to many other enzymes [15]. Due to these unique properties, lipases are the most attractive enzymes for use in various industrial applications, such as in the food processing industry [16,17] and in the energy industry for biodiesel production [18,19]. Protein engineering and optimization of lipase production systems make it possible to increase lipase productivity while decrease in product cost [20]. The production of active lipases has been performed in *Escherichia coli* [21], in *Saccharomyces cerevisiae* [22,23] and in *P. pastoris* [24-26]. Using *E. coli* as host encounter problems on lacking of post-transcriptional modification and formation of inclusion body and proteins expressed in *S. cerevisiae* are usually hyperglycosylated with high mannose glycans. *P. pastoris* expression system offers advantages of moderate glycosylation, tightly regulation, high level protein expression compared to *E. coli* and *S. cerevisiae*. Glycosylation of lipases expressed in yeast has multi-effects on their expression and properties. N-glycosylation on the lipases from *Rhizomucor miehei* was important for the secretion of the enzyme [27]. N-glycosylation of lipases from *R. miehei* and *R. oryzae* had a negative effect on the lipase activities [27,28]. The N-glycosylated *Thermomyces lanuginosus* lipase exhibited better thermostability than their non-glycosylated variants [29].

**Figure 1:**
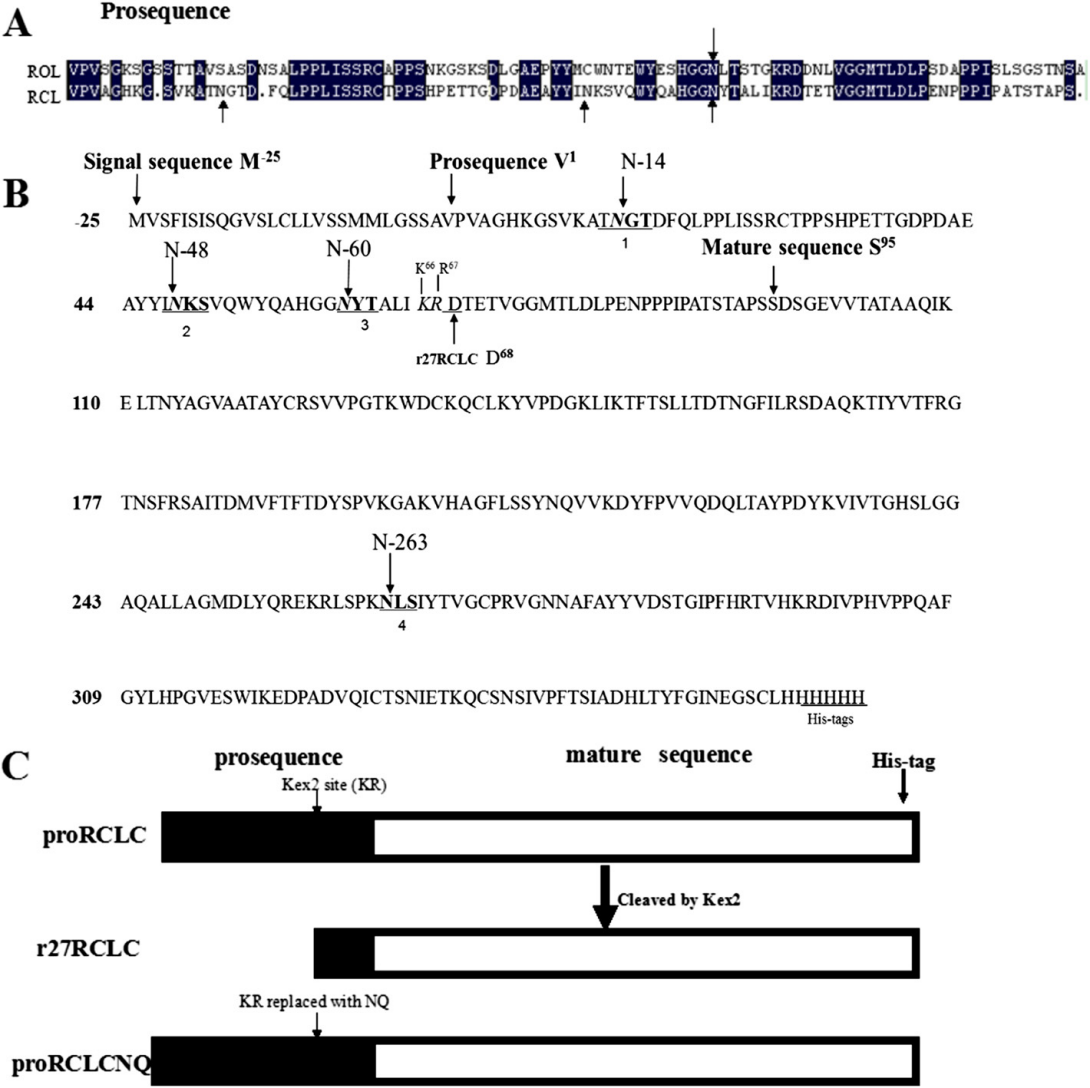
**Sequence analysis of the gene encoding** ***R. chinensis*** **lipase. A**. Alignment of the prosequence from *R. oryzae* lipase and *R. chinensis* lipase. Potential N-glycosylation sites were indicated by arrows; **B**. Amino acid sequence of *R. chinensis* lipase. All potential N-glycosylation sites of RCLC predicted through glycomod (http://web.expasy.org/glycomod/) were highlighted in bold font (N-14, N-48, N-60, N-263), and the initial amino acid of the signal sequence, prosequence and mature sequence were marked. The cleavage site-*K* ^*66*^ *R* ^*67*^ of Kex2 is indicated in italic font; **C**. Schematic of lipases proRCLCNQ and r27RCLC.

In our previous studies, the lipase gene from *Rhizopus chinensis* (GenBank accession no. EF405962) was cloned and expressed in *P. pastoris* [30]. A chimeric lipase from *R. oryzae* replaced with the prosequence from *R. chinensis* lipase (RCL) successfully expressed in *P. pastoris* at high-level, which was 11-fold higher than the wild type *R. oryzae* lipase (ROL) [31]. Three potential N-glycosylation sites are found in the propeptide of RCL, while ROL possesses only one potential N-glycosylation site in its prosequence (Figure 1A). These findings inspired us to explore whether N-glycosylation exists in the propeptide of RCL and how glycosylation affects the function of RCL. In this study, we generated a series of glycosylation mutants of RCL, by substituting the N-linking site with Q, and then we examined the expression levels of the N-glycosylation mutants. The roles of N-glycosylation on the protein expression, enzyme activity and thermostability of RCL were discussed.

## Results

### Sequence analysis of the gene encoding *R. chinensis* lipase

The RCL sequence contains one complete open reading frame without introns, which encodes a 389 amino acid protein including a 26 amino acid signal sequence, 94 amino acid prosequence and 269 amino acid mature lipase sequence (Figure 1B). As shown in Figure 1B, RCL has four potential N-linked glycosylation sites, three of which lie in the prosequence (N-14, N-48, N-60) and the fourth (N-263) is in the mature region. Because a Kex2 cleavage site at K^66^R^67^ is present in the prosequence, the *R. chinensis* prolipase (proRCLC) expressed in *P. pastoris* was truncated by Kex2 endoprotease. The resulting product was the mature lipase attached with 27 amino acids of the carboxy-terminal part of the prosequence, containing an his tag, named r27RCLC (Figure 1C), in which three potential N-glycosylation sites in the propeptide were removed, retained only one potential glycosyaltion site (N-263) in the mature region. After K^66^R^67^ in the prosequence was mutated into N^66^Q^67^, RCL expressed in *P. pastoris* was no longer cleaved by Kex2, which contains an entire prosequence and mature sequence, named proRCLCNQ (Figure 1C).

### Treatment of proRCLCNQ and r27RCLC with glycosidase

**Figure 2:**
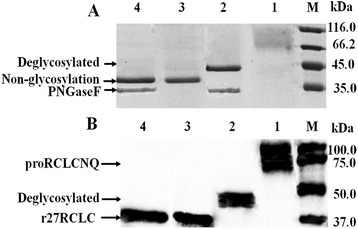
**Treatment of purified proRCLCNQ and r27RCLC by glycosidase. A**. SDS-PAGE analyses of purified proRCLCNQ and r27RCLC treated with PNGase F. **B**. Western blotting verification of proRCLCNQ and r27RCLC treated with PNGase F. Lane M, Marker; Lane 1, proRCLCNQ; Lane 2, proRCLCNQ treated with PNGase F; Lane 3, r27RCLC; Lane 4, r27RCLC treated with PNGase F.

The molecular weight of purified r27RCLC and proRCLCNQ were evaluated by SDS-PAGE and western blotting. The mass of r27RCLC (Figure 2A: Lane 3) was 37 kDa, which was higher than the calculated molecular weight of 32.27 kDa, while the mass of proRCLCNQ smeared between 66.2 kDa and 116 kDa (Figure 2A: Lane 1), which was much higher than the calculated molecular weight of 40.5 kDa [32]. After digestion with glycosidase PNGase F, SDS-PAGE analyses showed that the molecular mass of proRCLCNQ was reduced to approximately 45 kDa (Figure 2A: Lane 2), and the band was no longer smeared. Western blotting analyses confirmed that the band of proRCLCNQ (Figure 2B: Lane 2) treated with PNGase F shifted down, indicating that proRCLCNQ is a glycoprotein. In contrast, the mass of r27RCLC (Figure 2A: Lane 4) did not change in the SDS-PAGE after treatment with PNGase F, compared with the band of r27RCLC without treatment with glycosidase (Figure 2A: Lane 3). Western blotting analyses verified that the band of r27RCLC was the same before (Figure 2B: Lane 3) and after treatment with PNGaseF (Figure 2B: Lane 4), suggesting that r27RCLC was not N-glycosylated, that is the only potential N-glycosylation site at N-263 in the mature region was not glycosylated.

### Identification of the N-glycans in proRCLCNQ

**Figure 3:**
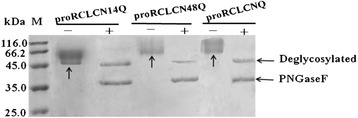
**SDS-PAGE analyses of purified proRCLCNQ and its N-glycosylation mutants treated with PNGase F.** (The smear bands of glycosylated protein were indicated by the arrows in the figure).

Each putative glycosylation site in the prosequence of proRCLCNQ was examined using site-directed mutagenesis from N (Asn) to Q (Gln). The N-glycosylation mutants of proRCLCNQ at the sites N-14, N-48 and N-60 were named proRCLCN14Q, proRCLCN48Q and proRCLCN60Q, respectively. Because protein proRCLCN60Q was not detected in the culture medium, we did not perform the treatment with glycosidase on it. SDS-PAGE was used to analyze the digestion of purified lipases with PNGase F (Figure 3). The mass of proRCLCN48Q did not change compared with proRCLCNQ, suggesting that the N-48 site was probably not glycosylated. The molecular mass of proRCLCN14Q, in which the glycosylation site at N-14 was deleted, showed a downward shift on SDS-PAGE compared with proRCLCNQ, indicating the site N-14 was glycosylated. After treated with PNGase F, the mass of proRCLCN14Q demonstrated an additional reduction to approximately 45 kDa and exhibited a single band (Figure 3), indicating that the site N-60 was glycosylated.

### Validation of the N-glycan in proRCLCNQ by LC-MS/MS analysis

**Figure 4:**
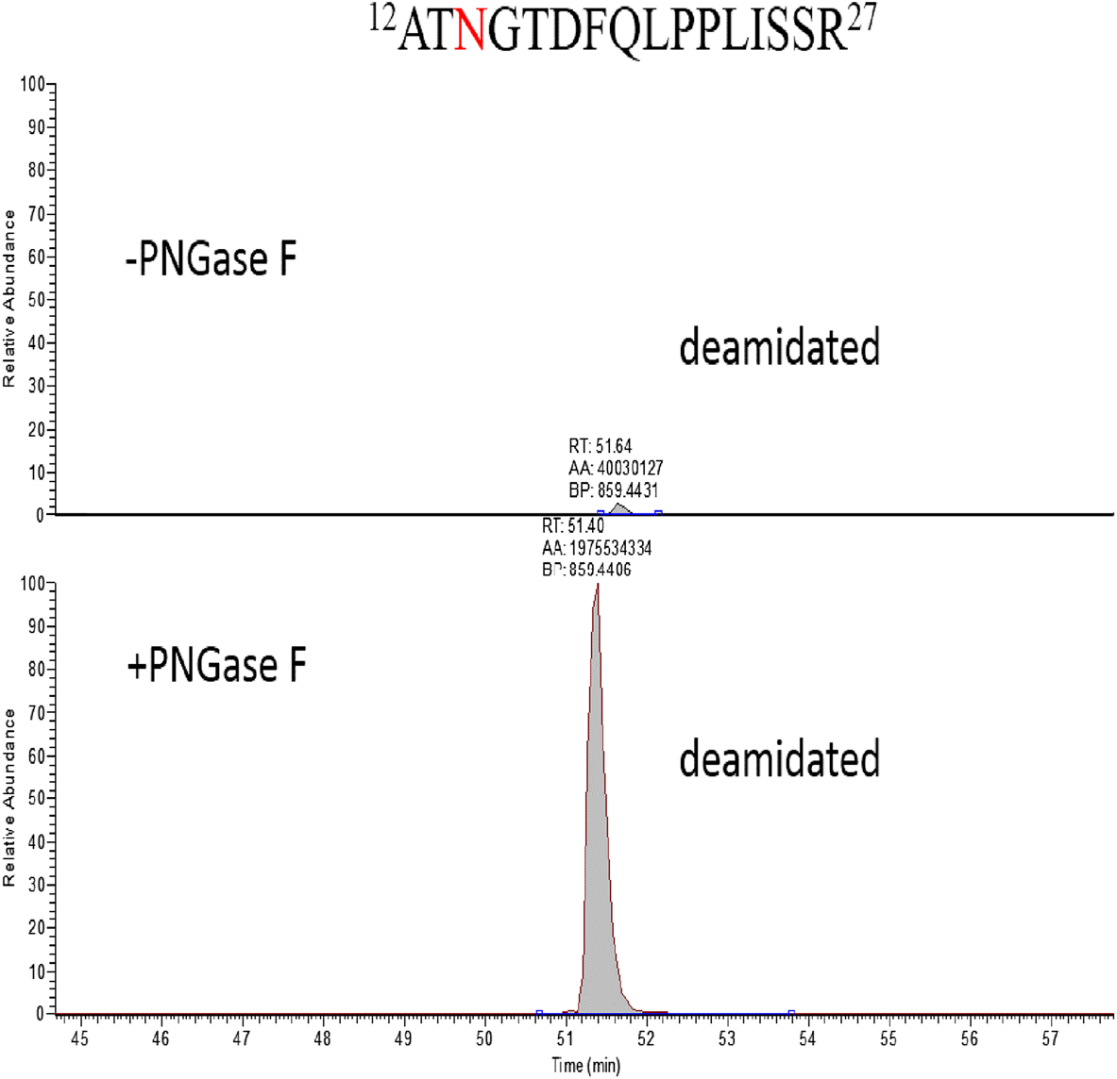
**Extracted ion chromatography of deamindated peptide** ^**12**^ **A-R** ^**27**^ **from proRCLCNQ.** The deamindated peptide ^12^ATN^14^GTDFQLPPLISSR^27^ was identified in tryptic digest before control (−PNGase F) or after treatment with PNGase F samples. The confirmed N-glycosylation site were marked in red. The automatic peak integration were indicated as AA. The peaks were scaled to 1.29E8 as 100% for both panels.

**Figure 5:**
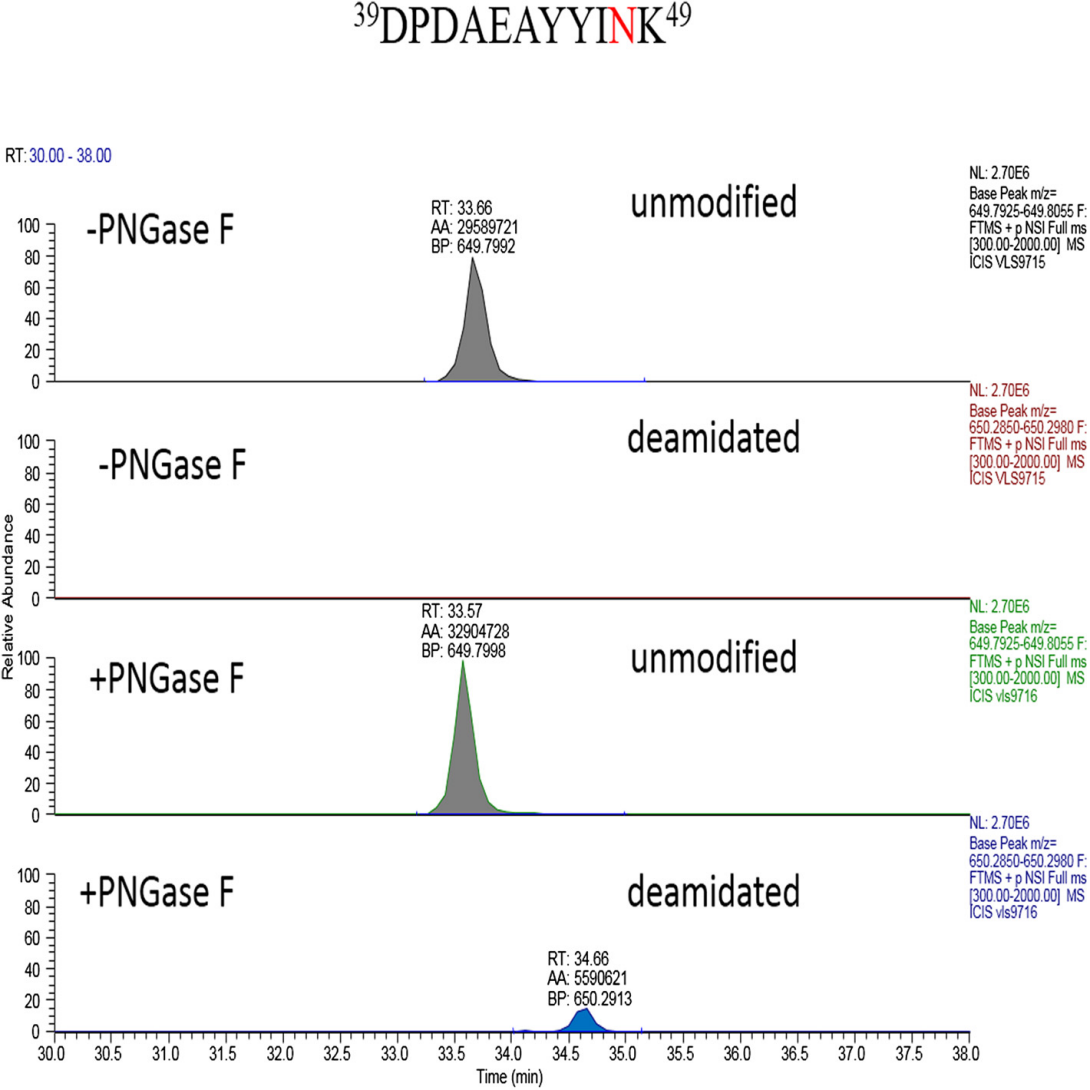
**Extracted ion chromatography of peptide** ^**39**^ **D-K** ^**49**^ **from proRCLCNQ.** The unmodified and deamindated peptides ^39^DPDAEAYYIN^48^K^49^ in control (−PNGase F) or after treatment with PNGase F samples obtained by AspN and trypsin double digestion were indicated in the figure. The confirmed N-glycosylation site were marked in red. The automatic peak integration of area under the curve were indicated as AA. The peaks were scaled to 2.7E6 as 100% for all panels.

**Figure 6:**
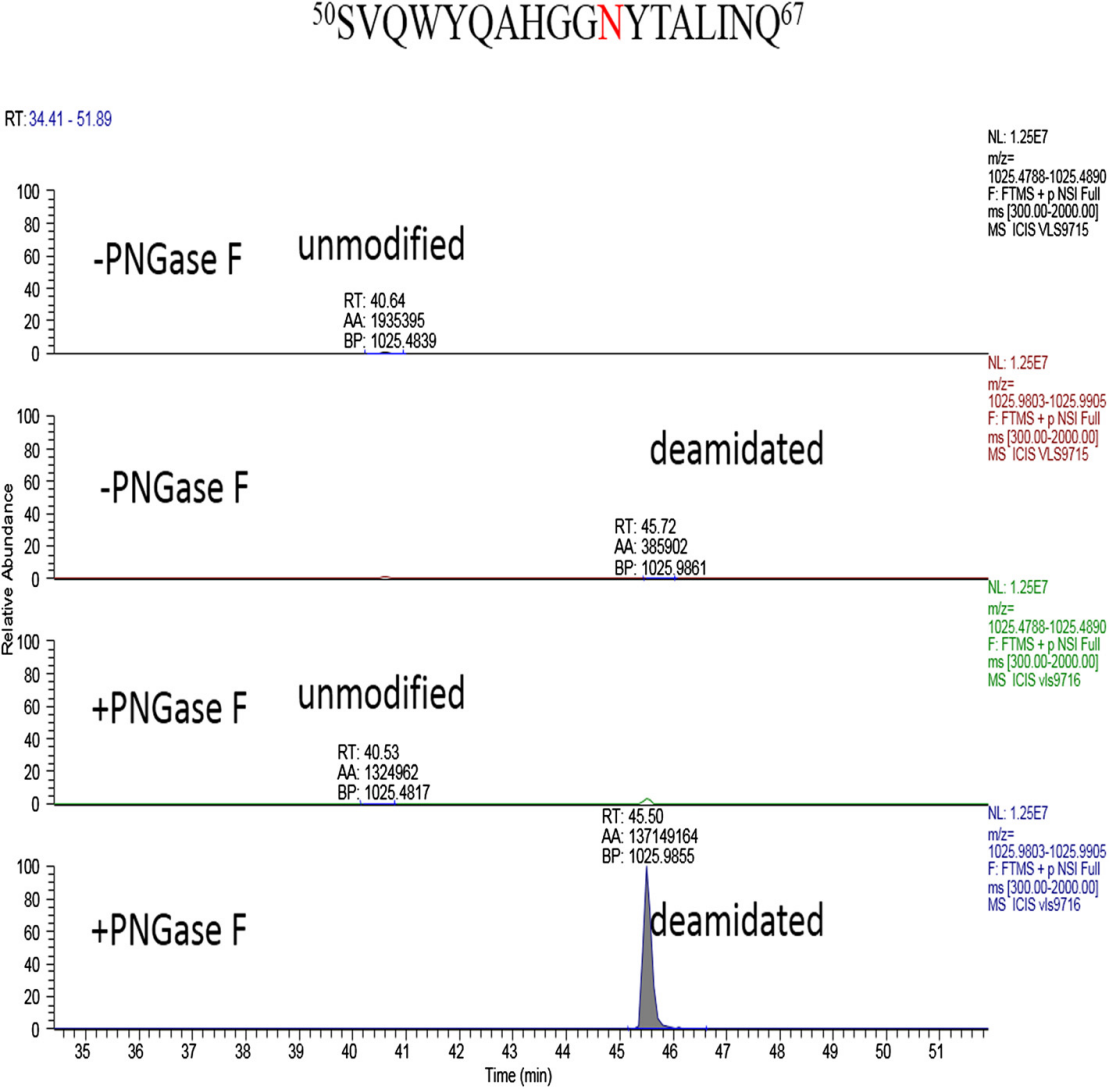
**Extracted ion chromatography of peptide** ^**50**^ **S-Q** ^**67**^ **from proRCLCNQ.** The unmodified and deamindated peptides ^50^SVQWYQAHGGN^60^YTALINQ^67^ in control (−PNGase F) or after treatment with PNGase F samples obtained by AspN and trypsin double digestion were indicated in the figure. The confirmed N-glycosylation site were marked in red. The automatic peak integration were indicated as AA. The peaks were scaled to 1.29E8 as 100% for both panels.

**Figure 7:**
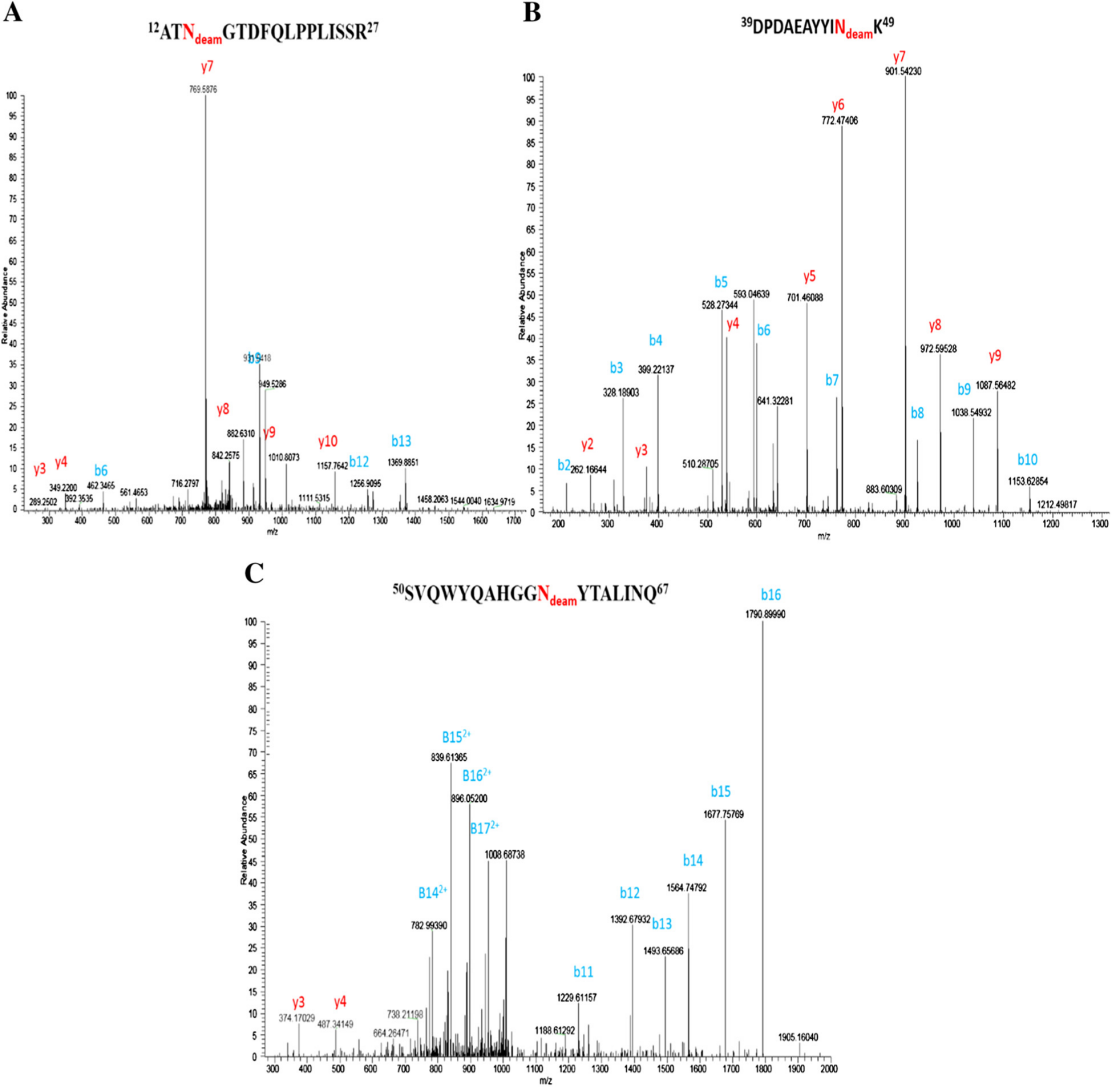
**LC-MS/MS tandem mass spectrum of deamindated peptide sequences obtained by enzymatic digestion. A**. the MS/MS spectrum of the deamindated peptide ^12^ATN_deam_GTDFQLPPLISSR^27^; **B**. the MS/MS spectrum of the deamindated peptide ^39^DPDAEAYYIN_deam_K^49^; **C**. the MS/MS spectrum of the deamindated peptide ^50^SVQWYQAHGGN_deam_YTALINQ^67^. Red residues in three peptides represent the N-glycosylation sites. The peptide sequence and the b and y type of fragments were indicated.

The extracted ion chromatography of the deamindated peptides ^12^A-R^27^ in control or treatment with PNGase F were shown in Figure 4. The spectra of the deamindated peptides ^12^A-R^27^ were significantly increased after treatment with PNGase F, indicating the significant amount of the sites N-14 in proRCLCNQ were N-glycosylated. In Figure 5, the extracted ion chromatography of the peptides ^39^D-K^49^ containing the site N-48 showed that the deamindated peptide (the second row) was slightly increased after treatment with PNGase F (the fourth row) and the majority of peptide (the first row) had no change after treatment with PNGase F (the third row), indicating only a small portion of the sites N-48 were glycosylated and the majority of the peptides were not modified. On the other hand, in Figure 6, the spectrum of deamindated peptide ^50^S-Q^67^ (the second row) was significantly increased after treatment with PNGase F (the fourth row), suggesting that the significant amount of the sites N-60 in proRCLCNQ were N-glycosylated. The MS/MS spectra of the three peptides mentioned above were shown in Figure 7. For the site N-263, LC-MSMS results certified that this site was not glycosylated (data not shown).

### Expression of N-glycosylation mutants in *P. pastoris*

**Figure 8:**
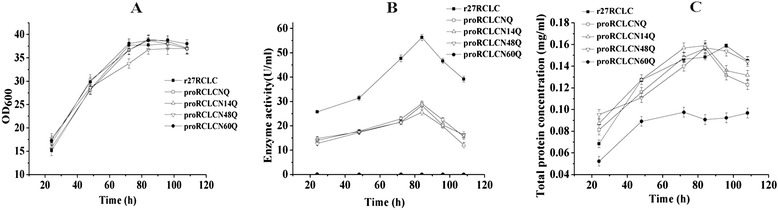
**Growth, extracellular enzyme activity and total protein concentration profiles of lipases. A**. Growth profiles of r27RCLC, proRCLCNQ and the N-glycosylation mutants; **B**. Extracellular enzyme activity of lipases. The curves were obtained from data of three independent experiments; **C**. Total protein concentration of lipases from culture supernatants. The curves were obtained from data of three independent experiments.

**Figure 9:**
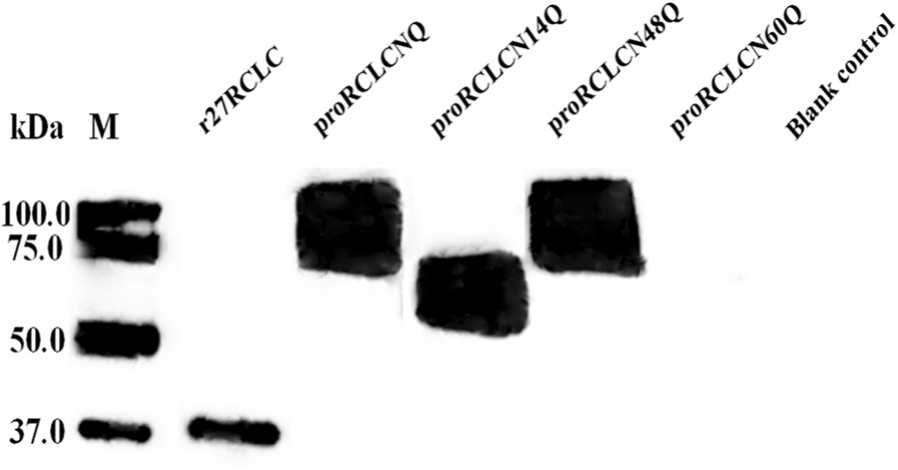
**Western blotting analyses of r27RCLC, proRCLCNQ and the N-glycosylation mutants from culture supernatant.**

To investigate the role of the carbohydrate chain attached to proRCLCNQ, we compared the extracellular secretion level and enzymatic activity of proRCLCNQ with its N-glycosylation mutants and the truncated r27RCLC in *P. pastoris.* All recombinant strains contained only one copy of the integrated lipase gene. The cell growth rates of all recombinant strains were comparable during the cultivation period (Figure 8A). As shown in Figure 8B, the enzyme activity of proRCLCN60Q was not detected, while those of proRCLCNQ, proRCLCN14Q and proRCLCN48Q were almost the same. However, compared with r27RCLC, the activities of proRCLCNQ and its mutants were much lower (Figure 8B). The kinetic assay (Table 1) also showed that the *k*_cat_ and *k*_cat_/*K*_m_ values for r27RCLC were the highest. On the other hand, the *k*_cat_ and *k*_cat_/*K*_m_ values for proRCLCNQ and proRCLCN48Q were very close, which were much higher than that of proRCLCN14Q losing the N-14 glycan. In Figure 8C, the total protein concentration of proRCLCN60Q was the lowest compared with others. Western blotting analyses (Figure 9) confirmed the bands of extracellular proRCLCNQ, proRCLCN14Q and proRCLCN48Q. In agreement with no activity detected for proRCLCN60Q, no Western blotting band was observed for this mutant. We further analyzed the transcription levels of the *r27RCLC*, *proRCLCNQ* and *proRCLCN60Q* genes by RT-PCR. Their transcription levels were almost the same at cultivation of 84 h, suggesting that the N-glycosylation mutation in the gene did not affect its transcription. We speculated that protein proRCLCN60Q was retained in yeast cells. Thus, the intracellular activity was measured and the intracellular lipase was analysis using Western blotting. Unfortunately, neither the intracellular N-glycosylation mutants (proRCLCN14Q, proRCLCN48Q, proRCLCN60Q) nor the parent proRCLCNQ could be detected on Western blotting.

**Table 1:** **Enzyme kinetic parameters of the purified r27RCLC, proRCLCNQ and its N-glycosylation mutants**

**Enzyme**	***k***_**cat**_ **(s** ^**−1**^ **)**	***K***_**m**_ **(mM)**	***k***_**cat**_ **/** ***K***_**m**_ **(M** ^**−1**^ **s** ^**−1**^ **)**
r27RCLC	121.54 ± 3.1	0.34 ± 0.10	(3.56 ± 0.81)*10^5^
proRCLCNQ	60.70 ± 2.2	0.20 ± 0.04	(2.92 ± 0.55)*10^5^
proRCLCN14Q	24.82 ± 2.4	2.01 ± 0.12	(1.22 ± 0.15)*10^5^
proRCLCN48Q	61.33 ± 2.5	0.19 ± 0.02	(3.11 ± 0.61)*10^5^

### Effects of N-glycan chains on enzyme stability

**Figure 10:**
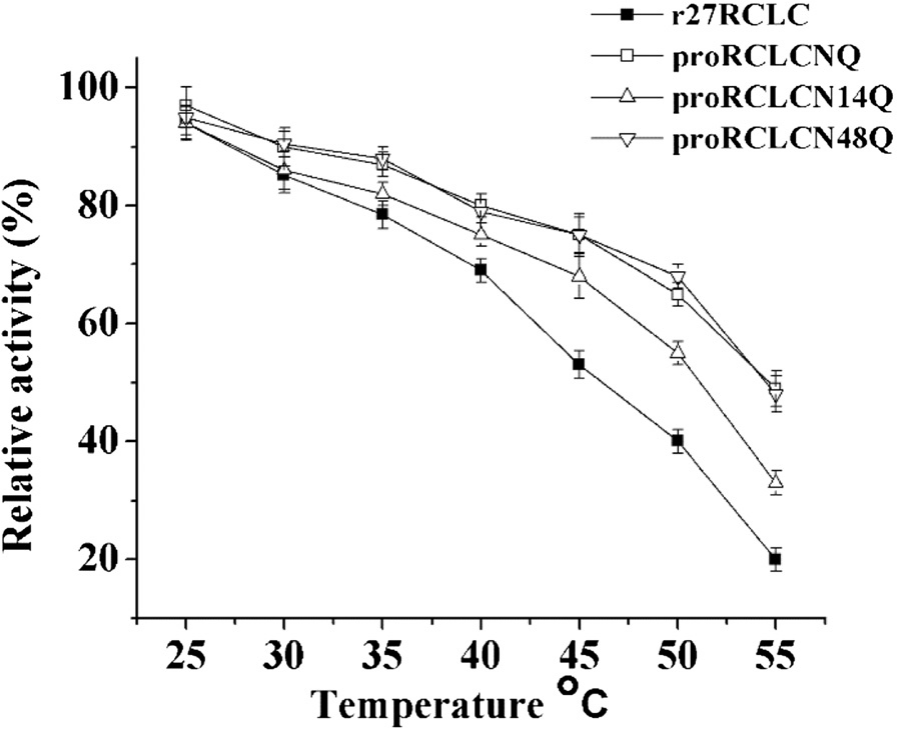
**Thermal stability of the purified r27RCLC, proRCLCNQ and the N-glycosylation mutants** **.** The samples were incubated for 1 h at different temperatures and the residual activities were measured.

To determine the effect of glycosylation on proRCLCNQ heat resistance, the thermostability of N-glycosylation mutants at different temperatures (25°C-55°C) retained 1 h was determined, and the results were illustrated in Figure 10. The lipases proRCLCNQ and proRCLCN48Q had about 70% residual activity after incubation for 1 h at 50°C, while the residual activity of r27RCLC was only 40% at the same time. On the other hand, the residual activity of proRCLCN14Q losing one N-glycan was lower than that of proRCLCNQ.

### Structural characteristics of secreted N-glycosylation mutants

**Figure 11:**
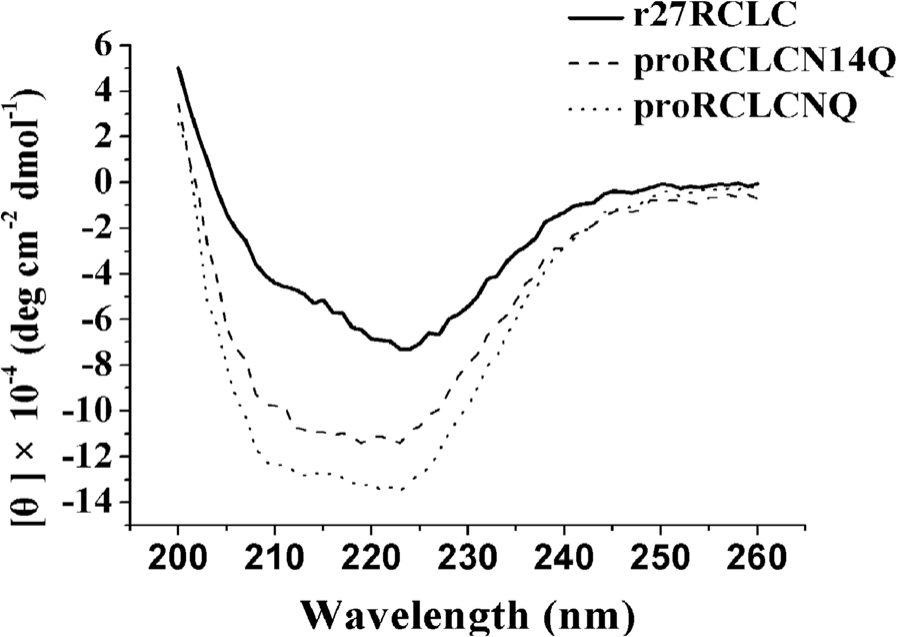
**CD spectra of the purified r27RCLC, proRCLCNQ and proRCLCN14Q.**

The structure of the secreted N-glycosylation mutants was characterized using CD spectra in the far-UV region (Figure 11). The CD spectrum of the glycoprotein proRCLCNQ showed a left-shift in wavelength range of 200–230 nm compared with r27RCLC. The spectrum of the mutant proRCLCN14Q mutant was similar to that of proRCLCNQ.

### Effects of organic solvents on lipase stability

The retained activities of enzymes after exposure to various organic solvents (90% (v/v)) at 20°C for 24 h were shown in Table 2. In the selected organic solvents, the relative activities of glycosylated proRCLCNQ and proRCLCN14Q were conspicuous higher than that of non-glycosylated r27RCLC. On the other hand, the residual activity of proRCLCN14Q losing one N-glycan was lower than that of proRCLCNQ. Furthermore, the relative activity of RCL in the presence of DMSO, *N*, *N*-dimethylformamide and dichloromethane was less than in the presence of hydrophobic solvents N-hexane and Isooctane. RCL may be more stable in the hydrophobic solvents than the polar solvents. These results indicated that the N-glycan may play a vital role for organic solvent-tolerance of RCL, especially solvents such as N-hexane and Isooctane.

**Table 2:** **The relative activities of r27RCLC, proRCLCNQ and proRCLCN14Q that treated by 90% (v/v) organic solvents with different Log** ***P*** **values**

**Organic solvent (90% v/v)**	**Log** ***P***	**Relative activities of lipases (%)**		
		**r27RCLC**	**proRCLCNQ**	**proRCLCN14Q**
None	-	100	100	100
DMSO	−1.49	29 ± 3.5	58 ± 4.0	51 ± 4.2
*N*, *N*-dimethylformamide	0.07	18 ± 4.3	43 ± 3.5	35 ± 3.1
Dichloromethane	1.01	8 ± 1.5	21 ± 2.1	16 ± 1.1
Toluene	2.50	22 ± 2.1	39 ± 2.5	31 ± 2.4
N-hexane	3.50	35 ± 3.5	66 ± 3.1	58 ± 3.2
Isooctane	4.50	43 ± 4.1	78 ± 3.5	70 ± 3.0

## Discussion

In this study, we examined the potential N-glycosylation sites of RCL, and then discussed the functional significance of N-glycosylation on its secretion and enzymatic properties. RCL has four potential glycosylation sites in its gene sequence, three of which lie in the prosequence and the fourth of which is in the mature sequence (Figure 1B). Although the potential N-glycosylation sites of a protein can be predicted from the consensus sequence Asn–Xaa–Ser/Thr, not all such sites are fully occupied [33]. When RCL was expressed in *P. pastoris*, its N-terminal was truncated by Kex2. Thus, the three potential glycosylation sites in its prosequence were removed and only one glycosylation site at N-263 was retained in the truncated lipase r27RCLC (Figure 1). Enzymatic deglycosylation, which removed both high-mannose, hybrid-and complex-type N-linked glycans, was performed using glycosidases to investigate whether the potential glycosylation sites were glycosylated or not [3]. Endo H_f_ cleaved within the chitobiose core of high mannose and some hybrid oligosaccharides from N-linked glycoproteins, leaving the innermost N-acetyl-glucosamine intact [34]. Peptide-N-Glycosidase F (PNGase F) is an amidase that cleaves between the innermost GlcNAc and asparagine residues of high mannose, hybrid, and complex oligosaccharides from N-linked glycoproteins [35]. On SDS-PAGE the band of r27RCLC digested with PNGase F were the same as the non-treated r27RCLC, indicating that the glycosylation site at N-263 was not glycosylated (Figure 2). Therefore, the site N-263 has no effect on the enzyme properties or the secretion of lipase. To investigate the effect of glycosylation in the prosequence of RCL, we constructed a mutant in which the Kex2 cleavage site-K^66^R^67^ of RCL was mutated into N^66^Q^67^ and subsequently expressed in *P. pastoris* GS115 to produce RCL containing its intact prosequence, named proRCLCNQ. To determine the contribution made to the molecular weight of recombinant proRCLCNQ by N-linked glycans, enzymatic deglycosylation was therefore performed using glycosidases. A reduction in the molecular mass of proRCLCNQ after treatment with glycosidase (Figure 2) demonstrated that proRCLCNQ is a glycosylated protein. The band of non-treated proRCLCNQ smeared and was higher than its calculated molecular weight. This discrepancy can be explained by the fact that varying conformations of the sugar moieties can alter the interactions with the electrophoresis gel or extent of SDS binding which finally results in an electrophoretic behavior which does not reflect the correct size of the corresponding protein [36]. Further studies by glycosidase digestion and nano-LC-MS/MS analysis of proRCLCNQ demonstrated that the majority of the sites N-14 and N-60 were glycosylated, while the glycosylation degree of the site N-48 was only a small portion. The very little amount of glycan on N-48 had no effect on the secretion and enzyme activity of RCL. However, after deletion of the glycan on N-60, the mutant proRCLCN60Q failed to be secreted into the supernatant (Figure 9) and also could not be detected in the cell extract. The result of RT-PCR showed that the transcription level of *proRCLCN60Q* was almost the same as that of *proRCLCNQ*, which suggested that the dramatic difference in the secretion level between proRCLCNQ and proRCLCN60Q was not caused by transcription. Christian *et al.* [7] confirmed that the glycan structures of the dirigent protein AtDIR6 in *P. pastoris* are essential for solubility, structure and function of the protein because deglycosylation induced conformational changes leading to the complete loss in dirigent activity and subsequent protein aggregation. In our experiment the N-60 glycan on proRCLCNQ may influence the proper translation or the correct folding of the enzyme, resulting proRCLCN60Q was not produced or the misfolded protein may be rapidly degraded, which could not be detected by Western blotting. Some scholars reported that glycans can play key roles in protein secretion or positioning. The glycosylation site in the peptide sequence of lysosomal renin represented the targeting signal that may as well serve for prorenin uptake via the mannose-6-phosphate receptor [37]. The N-glycosylation sites of the recombinant elastase were necessary for its high-level expression in *P. pastoris* [38]. Gwen *et al*. [39] demonstrated that human endothelial lipase was a glycosylated protein and that the efficient secretion of the enzyme was dependent on the presence of the N-linked carbohydrate. In our study, the glycan on N-60 may play a key role in positioning the protein to endoplasmic reticulum or transporting the protein from endoplasmic reticulum to Golgi apparatus. In future study, we plan to use fusion expression with GFP to observe the intracellular positioning of proRCLCN60Q for elucidation of its mechanism.

The secretion level of proRCLCN14Q was almost the same as proRCLCNQ (Figure 8C), which suggested that the glycan on N-14 had no effect on the secretion of this lipase. The *k*_cat_ value of proRCLCN14Q losing N-14 glycan apparently decreased (Table 1), which indicated that the glycan on N-14 probably assist in the protein folding, favoring a more beneficial conformation for higher activity. The important roles of N-glycan in enzyme activity have been reported. Wei *et al.* [8] demonstrated that the N-glycan on N-224 of β-Glucosidase in *P. pastoris* played a key role in protein native folding and catalytic activity. Kohler *et al*. [40] confirmed that the N-428 glycan of N-Acetylglucosamine-6-sulfotransferase-1 was critical for its enzyme activity.

The biomass and the extracellular total protein concentration of proRCLCNQ and proRCLCN14Q were very close to those of r27RCLC. However, r27RCLC showed the highest enzyme activity during cultivation (Figure 8B), which was in agreement with its highest *k*_cat_ and *k*_cat_/*K*_m_ values (Table 1). The existence of *Kex2* cleavage site in the wild-type RCL resulted in the partially truncation of the prosequence in Golgi apparatus, forming r27RCLC. The higher enzyme activity of the truncated r27RCLC indicated that the intact prosequence negatively affects the activity of RCL. The CD spectrum of glycoprotein proRCLCNQ showed a more classical α-helix structure in the wavelength range of 200–230 nm [6] compared with r27RCLC (Figure 11), which suggested that the intact prosequence altered the secondary structure of the lipase. Many proteins were synthesized in the form of precursor. After protein was folded into the mature form, its propeptide, an N-terminal peptide chain, were then identified and excised by the corresponding protease [41]. The function of propeptide is mainly divided into two categories. Type I is mainly responsible for the correct folding of the protein. These kinds of enzymes could only be activated after excision of their propeptides, for example, subtilisin [42] and lytic protease [43]. Type II is involved in intracellular protein transportation and positioning, not directly involved in protein folding, for example, somatostatin II [44] and myeloperoxidase [45]. Our results revealed that the propeptide in RCL probably acts not only as the type I but also as the type II propeptide and the N-glycan in the propeptide plays a key role.

In general, glycoproteins are more stable than their corresponding non-glycosylated counterparts, despite the lack of major structural changes associated with glycosylation [46]. Steric interactions between the sugar residues and protein structure have been reported to be involved in stabilizing the effects in many glycosylated proteins [47]. However, the sugar chains bound to *R. niveus lipase* [48] and *R. oryzae* lipase [28] had no effect on thermal stability, which demonstrated the different roles of sugar chains in the enzyme properties. Functionally active N-glycosylation mutants of proRCLCNQ enabled the study of the effect of glycosylation on protein stability. The results demonstrated that glycoprotein proRCLCNQ, proRCLCN48Q and proRCLCN14Q were more thermostable than r27RCLC (Figure 10). The covalent addition of the glycans to the surface of proRCLCNQ may modulate its kinetic parameters and thermal stability due to the interactions between the protein and the attached glycans, demonstrating that glycosylation affects the protein energy landscape [49]. After the deletion of the glycan on N-14, compared with proRCLCNQ, the thermal stability of proRCLCN14Q decreased. The glycan on N-14 of proRCLCNQ may improve the stability of the enzyme conformation by decreasing flexibility or adding rigidity to the enzyme structure [50,51]. On the other hand, the stability measurement of RCL in aqueous-organic mixtures suggested that the N-glycans on RCL improved the enzyme stability in organic solvents. The effect of glycosylation on enzyme stability in organic solvents has rarely been investigated. Enzyme tolerance to organic solvents differed from lipase to lipase [52]. Zou *et al*. demonstrated the N-glycans of *ß*-glucuronidase increased its stability in DMSO and acetone [53]. The stability of the glycosylated lipase proRCLCNQ from *P. pastoris* in organic solvents made it hold the potential for its use in organic synthesis and related applications. And we also measured the substrate specificity of r27RCLC, proRCLCNQ and proRCLCN14Q towards *p*-Nitrophenyl monoesters (C2 ~ C16). The results showed that the glycans on protein proRCLCNQ have no effect on the lipase substrate specificity (Data not shown).

## Conclusions

In summary, this study demonstrated that RCL is N-glycosylated when expressed in *P. pastoris* and confirmed the key role of the N-glycosylation in the secretion, enzyme activity and stability of RCL. This report may also provide theoretical support for the improvement of enzyme expression and stability based on the N-linked glycosylated modification to meet the future needs of the biotechnological industry and provide excellent biological catalyst for the oil processing industry and other biotechnological industries.

## Material and methods

### Enzymes and reagents

Endo H_f_ and PNGase F were purchased from New England BioLabs. *p*-nitrophenyl palmitate (*p*NPP), endoproteinase Asp-N and trypsion were obtained from Sigma (USA). Horseradish peroxidase-conjugated goat anti-mouse IgG, Anti-His antibody and Pro-LightHRP chemical reflective detection reagents was purchased from TianGen Biotech (Beijing, China). Western blotting Marker and nitrocellulose membrane (PVDF) were obtained from BIO-RAD. *Dpn* I, PrimeSTAR polymerase, PCR reagents were obtained from Takara Biotechnology (Dalian, China). SDS-PAGE Protein Marker was provided by Beyotime Institute Biotechnology. Primers were synthesized at Sangon Bitech (Shanghai, China). Gel extraction and PCR purification kits were purchased from Bioflux (Hangzhou, China). A Plasmid Mini Kit I was obtained from OMEGA Bio-Tek. A One Step Yeast Active Protein Extraction Kit was purchased from Sangon Bitech. All other chemicals used were of the highest quality that is commercially available.

### Strains and plasmids

*P. pastori*s GS115 and Plasmid pPIC9K were used as gene expression vector and purchased from Invitrogen. The constitutive recombinant plasmid pGAPK-*proRCLC* and the strain GS115/pGAPK-*proRCLC* expressing *R. chinensis* lipase were previously constructed in our lab [54]. Yeast nutrient medium MD-G418 and YPD-G418 were prepared using ‘*P. pastoris* expression Kit’ (*Pichia* Multi-Copy Expression Kit, version A, Invitrogen BV, The Netherlands).

### Construction of the recombinant *R. chinensis* lipase

The mutation of the Kex2 cleavage site in RCL from K^66^R^67^ to N^66^Q^67^ was generated by point mutation using synthetic oligonucleotide primers (5′-CTACACTGCTCTTATCAACCAGGATACTGAAACCGTCG-3′ and 5′-CGACGGTTTCAGTATCCTGGTTGATAAGAGCAGTGTAG-3′) with the plasmid pGAPK-*proRCLC* as the template. After the template was digested with *Dpn* I, the plasmid, named pGAPK-*proRCLCNQ* was transformed into *E. coli* JM109 competent cells as described by Hanahan [55]. Subsequently, the transformants were selected on an LB agar plate with ampicillin. After the mutation was verified using DNA sequencing, the recombinant plasmid pGAPK-*proRCLCNQ* was linearized with *Bgl* II and then transformed into *P. pastoris* GS115 competent cells by electroporation. The transformed cells were grown on an MD plate with G418 and cultured for the production of recombinant lipase named proRCLCNQ.

### Construction of N-glycosylation mutants

Mutations in each of the glycosylation sites were generated by a point mutation as previously described, using the plasmid pGAPK-*proRCLCNQ* as the template. For each glycosylation site, the codon for N (Asn) was replaced by Q (Gln) at position 14, 48 and 60, respectively. The following sense oligonucleotides were used to generate the mutant of proRCLCN14Q, proRCLCN48Q and proRCLCN60Q, individually:

- 5′-GTTCAGTCAAGGCAACTCAGGGCACCTTTGAC-AACTC-3′;
- 5′-AGCTTACTATATTCAGAAGAGCGTTCAATGTACCAAG-3′;
- 5′-CAATGGTACCAAGCTCACGGTGGCCAGTACACTTCTTATCAACC-3′.

The methods for transformation and expression were the same as the procedure described above. All lipases in this study were constructed with a six-histidine tag at the C-terminus.

### Expression in *P. pastoris* in shaking flasks

The *P. pastoris* transformants were cultured in 100 ml of YPD medium shaken at 30°C and 200 rpm in 500 ml glass flasks. The culture supernatant was collected every 12 h or 24 h to assay the cell density, protein concentration and lipase activity during culture.

### Determination of the extracellular secretion level and lipase activity

Lipase activity was measured on emulsified *p*NPP according to Kordel *et al*. [56].

One volume of a 1.08 mM solution of *p*NPP in 2-propanol was mixed just prior to use with nine volumes of 50 mM PBS buffer pH 8.0, containing 4 g/L Triton X-100 and 1 g/L arabic gum. The standard reaction was 2.4 mL of the above substrate mixture and 0.1 mL of enzyme solution at an appropriate dilution in 50 mM pH 8.0 PBS buffer at 40°C for 2 min. The absorbance at 410 nm of the reactant against a blank without enzyme was monitored using a UV–vis spectrophotometer (UNICO UV-3102 PC, China). One enzyme unit was defined as the amount of enzyme releasing 1 μmol of *p*-nitrophenol per minute under the assay conditions (pH 8.0, 40°C). SDS-PAGE and Western blotting analyses were used to analyze the secretion level of the mutants. The protein concentration was determined using a Bradford assay. Bovine serum albumin (BSA) was used as a standard.

### Analyses of the intracellular expression level of N-glycosylation mutants

For the assays of intracellular protein and activity, cells expressing each of the N-glycosylation mutants were harvested every 12 h from 24 h until 96 h during culture and were separated by centrifugation (7000 × g for 10 min). Next, the cells were lysed using a One Step Yeast Active Protein Extraction Kit, which consisted an extraction reagent, a protease inhibitor, DTT and PMSF solution. The lysates were used to analyze the lipase activity and were subjected to Western blotting analyses to monitor intracellular proteins.

### Transcription level of the lipase gene

The transcription levels of the lipase genes in the constructed recombinant strains after cultivation of 84 h were analyzed using reverse transcription-polymerase chain reaction (RT-PCR) normalized with *Actin* gene as the housekeeping gene. RNA was isolated using Yeast RNAiso Kit (TaKaRa Bio Co., Ltd). RNA integrity was tested in 1.2% agarose gels and its concentration was measured by densitometry and by 260/280 nm absorbance ratio. Five hundred nanograms of total RNA were subjected to reverse transcription using AMV First Strand cDNA Synthesis Kit (Sangon Bio Co., Ltd). The reaction was terminated by heating at 70°C for 10 min. Synthetic primers used in RT-PCRs are as follows. Actin-F: 5′-TGGTAACGAAAGATTCAGAGCC -3′, Actin-R: 5′-TGATGGAGTTGTAAGTAGTTTGGTC-3′; Target-F:

- 5′-GGTTGTCCTCGTGTCGGTAA-3′,
- Target-R: 5′-GATTTGAACATCAGCAGGGTCT-3′

Samples were run in triplicate in optical 96-well PCR plates with values falling within ±1%. Running conditions included 25 μl final volume, 25 μl 2 × SYBR mix, 1–100 ng DNA, and 10 μM reverse and forward primers. Control samples included a minus RT sample to ensure that there was no DNA contamination. Analysis was performed using a comparative (ΔC_t_) approach. Additionally, a standard curve using dilutions of the sample was created.

### Purification of lipases

The selected yeast strain was grown in 100 ml YPD medium for 72 h, and then the culture medium was centrifuged (7000 × g, 30 min) to remove cells. The histidine-tagged lipases from the culture supernatant were purified using Ni-NTA chromatography by ÄKTA purifier (GE Co.). Lipases were then concentrated by ultrafiltration through a 10-kDa membrane (Millipore, USA). The purity of the proteins was monitored using SDS-PAGE. The purification methods of all lipases mentioned in the paper were the same as above.

### SDS-PAGE and Western blotting analyses

Denaturing SDS-PAGE was performed as previously described by Laemmli [57]. Protein samples were subjected to 12% SDS–PAGE using a Mini-Protein II Cell (Bio-Rad). Proteins were stained with Coomassie bright blue and quantified using a Molecular Imaging System, with the low protein ladder (Takara, china) as a standard. For Western blotting analysis, the proteins were separated using electrophoresis and then transferred onto a Protran nitrocellulose membrane using a Mini Trans-Blot Cell (Bio-Rad). A purified Anti-His Antibody raised against the purified RCL was used as the primary antibody and was diluted 1:1,000 prior to application. Horseradish peroxidase-conjugated goat anti-mouse IgG was diluted 1:500 as the secondary antibody. An immunoblot assay system (Bio-Rad Laboratories) was used to quantify the relative amount of protein.

### Glycosidase digestions

Protein samples (0.025 mg/ml) were boiled for 10 min in denaturing buffer containing 0.4 M DTT and 0.5% SDS to fully expose all of the glycosylation sites, and deglycosylation was then performed by treatment with Endo H_f_ or PNGase F at 37°C for overnight according to the instructions of the manufactures. The buffers used in these enzyme reactions were 50 mM sodium citrate (pH 5.5) for EndoH_f_ and 50 mM sodium phosphate (pH 7.5) containing 1% Nonidet P-40 for PNGase F. The supernatant (20 μl) of each culture medium was subjected to SDS-PAGE and Western blotting analyses.

### In-gel digestion

5 μg of protein were separated on SDS-PAGE and the visible bands were excised and combined for in-gel tryptic digestion. Standard protocol was used for reduction, acylation and in-gel tryptic digestion and peptide extraction. For PNGase F treatment, in-gel digested peptides were divided to 2 equal aliquots and solubilized in 50 mM NH_4_HCO_3_. One aliquot was digested with PNGase F at 37°C for 2 hours (+PNGase F) and the other aliquot was incubated alongside without PNGase F as control (−PNGase F). The reaction was stopped by addition of 10% TFA to adjust pH to 3 before LC-MS/MS.

### LC-MS/MS and data analysis

LC-MS/MS was done using nano-LC-MS/MS using a Dionex RSLC system (ThermoFisher, San Jose, CA) interfaced with a LTQ Orbitrap Velos (ThermoFisher, San Jose, CA). Samples were loaded onto a self-packed 100 μm × 2 cm trap packed with Magic C18AQ, 5 μm 200 A (Michrom Bioresources Inc, Aubum, CA) and washed with Buffer A( 0.2% formic acid) for 5 min with flow rate of 10 μl/min. The trap was brought in-line with the homemade analytical column (Magic C18AQ, 3 μm 200 A, 75 μm × 50 cm) and peptides fractionated at 300 nl/min with a multi-stepped gradient (4 to 15% Buffer B (0.16% formic acid 80% acetonitrile) in 10 min and 15-50% B in 40). Mass spectrometry data was acquired using a data-dependent acquisition procedure with a cyclic series of a full scan acquired in Orbitrap with resolution of 60,000 followed by MS/MS scans (CID 35% of collision energy) of 20 most intense ions with a repeat count of two and the dynamic exclusion duration of 60 sec. The LC-MS/MS data was searched against a custom fasta database including target protein sequences using X!tandem (SLEDGEHAMMER (2013.09.01), thegpm.org) with carbamidomethylation on cysteine as fixed modification and oxidation of methionine and deamidation on Asparagine as variable modifications using a 10 ppm precursor ion tolerance and a 0.4 Da fragment ion tolerance. Relevant peptides were manually inspected. Glycopeptides were interpreted by manual inspection of the LC-MSMS raw data.

### Kinetic parameters

The kinetic parameters of purified enzymes were determined under the react condition of pH 8.0 and 40°C using various concentrations of *p*NPP as substrate according to the method described by Burdette *et al*. [58].

### Thermostability analysis

The purified lipases were incubated for 1 h at different temperatures and then the residual enzyme activities of lipases were measured at 40°C using *p*NPP as substrate by the standard enzyme activity detection method as previously described [56].

### Lipase treated with organic solvents

The organic solvents were selected and classified according to their Log *P* values [59]. Lipase was incubated in 90% (v/v) of DMSO, *N, N*-dimethylformamide, dichloromethane, toluene, *n*-hexane and isooctane at 20°C and 200 rpm for 24 h. Stability of lipases in these organic solvents was probed by measuring the residual activity of the mixture, using *p*NPP as substrate as previously described. The mixtures of lipases and PBS buffer (50 mM, pH 8.0) were taken as control. The activity of the control was taken as 100%.

### Circular Dichroism (CD) spectra

CD spectra were taken on a MOS-450/AF-CD (Jasco), which was continuously purged with nitrogen. Measurement was performed at 25°C for a final concentration of 0.2 mg/ml in 10 mM potassium phosphate buffer (pH 8.0) using the cell with 1.0 mm pathlength for far-ultraviolet CD spectra (200–260 nm). An average of three consecutive scans was taken for each sample. Each spectrum was represented as the mean residue ellipticity (degree cm^−2^ dmol^−1^).

## Abbreviations

RCL: *Rhizopus chinensis* lipase
ROL: *Rhizopus oryzae* lipase
r27RCLC: *Rhizopus chinensis* mature lipase attached with 27 amino acids of the carboxy-terminal part of the prosequence
proRCL: *Rhizopus chinensis* lipase with intact prosequence
proRCLCNQ: *Rhizopus chinensis* lipase with intact prosequence with K^66^R^67^ mutated into N^66^Q^67^
proRCLCN14Q: The N-glycosylation mutant of proRCLCNQ at the site N-14
proRCLCN48Q: The N-glycosylation mutant of proRCLCNQ at the site N-48
proRCLCN60Q: The N-glycosylation mutant of proRCLCNQ at the site N-60
YPD: Yeast extract peptone dextrose medium
GFP: Green fluorescent protein
Endo H_f_: Endoglycosidase H_f_
PNGase F: Peptide: N-glycanase F
*p*NPP: *p*-nitrophenyl palmitate
